# Draft Genome Sequence of Multidrug-Resistant Stenotrophomonas pavanii BWK1, Isolated from Mareca penelope Feces

**DOI:** 10.1128/genomeA.00187-18

**Published:** 2018-03-22

**Authors:** Takehiko Kenzaka, Katsuji Tani

**Affiliations:** a Environmental Science and Microbiology, Faculty of Pharmacy, Osaka Ohtani University, Tondabayashi, Japan

## Abstract

Migratory birds serve as vectors by transmitting antibiotic-resistant bacteria across large distances. Here, we isolated a multidrug-resistant Stenotrophomonas pavanii strain, BWK1, from Mareca penelope feces. Analysis of the draft genome sequence of the isolated strain indicated that BWK1 harbors a class A beta-lactamase, metallo-beta-lactamase, and several multidrug efflux pumps.

## GENOME ANNOUNCEMENT

Bacteria of the genus Stenotrophomonas are found in a wide range of ecosystems. These bacteria exhibit degradation capabilities and the potential for biotechnological applications as well as clinical relevance (1). Currently, at least 17 species of the genus Stenotrophomonas are recognized, which were isolated from various environmental sources (2). Stenotrophomonas pavanii was first isolated from sugarcane stems in 2011 (3). However, the ecology and pathogenesis of S. pavanii remain poorly understood.

Multidrug-resistant S. pavanii BWK1 was isolated on the CHROMagar extended-spectrum beta-lactamase (ESBL) medium (Kanto Chemical Co., Inc., Tokyo, Japan) from Mareca penelope feces. BWK1 showed resistance to carbapenems and aminoglycosides. M. penelope (Eurasian wigeon) is a migratory bird that primarily inhabits lakes, rivers, and coastlines, preferring areas of water with land plants nearby to feed upon in Eurasia and North America (4).

The draft genome sequence of S. pavanii BWK1 was analyzed using 100-bp paired-end sequencing on the Illumina HiSeq 2000 sequencing system (Hokkaido System Science Co., Ltd., Sapporo, Hokkaido, Japan). High-quality sequence reads (45,933,011) were assembled *de novo* using CLC Genomics Workbench (version 6.5; CLC bio, Cambridge, MA, USA). Approximately 99.7% of the sequenced reads were remapped to the contigs. The final assembly of the genome produced 4,403,137 bp in 45 contigs, with an *N*_50_ value of 288,044 bp and a GC content of 67.4%. The assembled contigs were functionally annotated using the Rapid Annotations using Subsystems Technology (RAST) server (5). The genome comprised 3,980 putative coding sequences and 69 RNA genes.

The BWK1 genome encoded the L2 family class A beta-lactamase and subclass B3 metallo-beta-lactamase. These class A and B beta-lactamases encoded on the chromosome are conserved in other S. pavanii and Stenotrophomonas maltophilia strains (6). In addition, the BWK1 genome encoded the multidrug resistance proteins aminoglycoside N6ʹ-acetyltransferase, multidrug resistance tripartite systems, resistance-nodulation-division (RND) efflux system membrane fusion protein/inner membrane transporter/outer membrane lipoprotein (CmeA, CmeB, and CmeC), multidrug and toxin extrusion family efflux pump, macrolide-specific efflux protein MacA, macrolide export ATP-binding/permease protein MacB, and membrane fusion protein of the RND family multidrug efflux pump. Stenotrophomonas species are known to show resistance against a broad range of antibiotics (1, 7). There are many resistance genes against various antibiotics in the genome of S. pavanii BWK1. Moreover, this genome encoded the biosynthesis and the sensor-receptor systems of siderophore, hemolysin, hemolysin secretion protein, and the type II and IV secretion systems, which are important virulence factors (8–10).

Taken together, our results suggest that M. penelope can transmit multidrug-resistant S. pavanii from birds to humans and *vice versa* through their migration between eastern Asia and eastern Siberia. The genome analyses of S. pavanii BWK1 will facilitate the understanding of the ecology and the global distribution of S. pavanii transmitted by migratory birds (1, 3). Studies regarding the association between S. pavanii and M. penelope may help improve the understanding of the dissemination of antibiotic resistance in the environment.

### Accession number(s).

The draft genome sequences of the S. pavanii strain BWK1 have been deposited in the DDBJ/EMBL/GenBank with the accession number PPHR00000000.
